# Diurnal Rhythms of Bone Turnover Markers in Three Ethnic Groups

**DOI:** 10.1210/jc.2016-1183

**Published:** 2016-06-13

**Authors:** Jean Redmond, Anthony J. Fulford, Landing Jarjou, Bo Zhou, Ann Prentice, Inez Schoenmakers

**Affiliations:** Medical Research Council (MRC) Human Nutrition Research (J.R., A.P., I.S.), Cambridge CB1 9NL, United Kingdom; MRC Keneba (A.J.F., L.J., A.P.), Banjul, The Gambia; MRC International Nutrition Group (A.J.F.), London School of Hygiene & Tropical Medicine, London WC1E 7HT, United Kingdom; and Department of Public Health (B.Z.), Shenyang Medical College, Shenyang 110034, People's Republic of China

## Abstract

**Context:**

Ethnic groups differ in fragility fracture risk and bone metabolism. Differences in diurnal rhythms (DRs) of bone turnover and PTH may play a role.

**Objective:**

We investigated the DRs of plasma bone turnover markers (BTMs), PTH, and 1,25(OH)_2_D in three groups with pronounced differences in bone metabolism and plasma PTH.

**Participants:**

Healthy Gambian, Chinese, and white British adults (ages 60–75 years; 30 per country).

**Interventions:**

Observational study with sample collection every 4 hours for 24 hours.

**Main Outcomes:**

Levels of plasma C-terminal telopeptide of type I collagen, procollagen type-1 N-propeptide, N-mid osteocalcin, bone alkaline phosphatase, PTH, and 1,25-dihydroxyvitamin D were measured. DRs were analyzed with random-effects Fourier regression and cross-correlation and regression analyses to assess associations between DRs and fasting and 24-hour means of BTMs and PTH.

**Results:**

Concentrations of BTMs, PTH, and 1,25-dihydroxyvitamin D were higher in Gambians compared to other groups (*P* < .05). The DRs were significant for all variables and groups (*P* < .03) and were unimodal, with a nocturnal peak and a daytime nadir for BTMs, whereas PTH had two peaks. The DRs of BTMs and PTH were significantly cross-correlated for all groups (*P* < .05). There was a significant positive association between C-terminal telopeptide of type I collagen and PTH in the British and Gambian groups (*P* = .03), but not the Chinese group.

**Conclusions:**

Despite ethnic differences in plasma BTMs and PTH, DRs were similar. This indicates that alteration of rhythmicity and loss of coupling of bone resorption and formation associated with an elevated PTH in other studies may not uniformly occur across different populations and needs to be considered in the interpretation of PTH as a risk factor of increased bone loss.

## Abstract (precis)

Diurnal rhythms of bone turnover markers and parathyroid hormone were similar among three ethnic groups in the presence of pronounced differences in their 24h mean concentrations.

Ethnic groups differ in fragility fracture risk, bone mineral density (BMD), and the rate of bone turnover (1–3). The etiology of these differences is multifactorial and results from complex interactions between genetic traits, environmental and lifestyle factors, hormone status, and patterns of secretion. African Americans are reported to have higher plasma PTH concentrations and a concomitant lower rate of bone turnover compared to white Americans (2–5), suggesting that there may be differences in the sensitivity to the bone-resorbing effects of PTH between these groups (6). This is thought to partly explain the relatively high BMD and low fracture rates in African Americans. In contrast, in The Gambia plasma concentrations of PTH, 1,25-dihydroxyvitamin D [1,25(OH)_2_D] and bone turnover markers (BTMs) are higher compared to white British subjects throughout life (7–9). A relative skeletal resistance to PTH in Gambians compared to white British subjects was not found (7). Although Gambians have a relatively low BMD on a population basis, the prevalence of fragility fractures is low (10, 11). Similar findings were reported in older Chinese people, although less pronounced (7, 12). In this population, the negative relationship between PTH and bone mineral content as found in older white people is absent, and the risk of osteoporosis and fragility fractures is lower than in white British older adults (12, 13).

The process of bone remodeling exhibits pronounced daily rhythmicity, which is reflected in plasma BTM concentrations (14–17). This rhythmicity is thought to be partly regulated by PTH and may be modulated by external factors, such as eating patterns (17–19). These rhythms should thus be considered as diurnal (rhythm having daily cycles under habitual conditions, which may or may not have an underlying endogenous circadian component; the latter is defined as those daily cycles of physiology and behavior that persist independent of external influences) (20). Osteoporosis and secondary hyperparathyroidism are associated with a different diurnal rhythm (DR) of PTH and BTMs compared to healthy controls, suggesting that the DRs of PTH and bone remodeling are important for bone integrity (19, 21–23). The importance of circadian clock genes in the regulation of bone metabolism and bone volume is further illustrated in alterations of these in mice with a single mutant for these genes (24, 25).

Little is known about racial or ethnic differences (as defined elsewhere) (26) in DRs of bone remodeling and whether these are related to the known differences in bone metabolism between these groups. Only one study investigated ethnic differences in the DR of PTH and bone resorption (27). In this study with healthy premenopausal African American and white American women, the 24-hour mean plasma PTH was higher in African American women, and urinary amino-terminal collagen crosslinks and deoxypyridinoline per unit urinary creatinine were comparable. The nocturnal increase in PTH observed in white women was absent in African American women, whereas in both groups a similar nocturnal increase in urinary excretion of bone resorption markers was found. It was suggested that these differences may contribute to their differences in net calcium conservation and BMD (27).

Here we report differences in the DRs of BTMs and their regulators in older adults of three distinct ethnic groups: Gambian, Chinese, and white British. They differ in lifestyle, dietary patterns (particularly calcium intake), and environmental influences, modulating fasting concentrations of calciotropic hormones, rates of bone turnover, and vitamin D status. We investigated these three groups during a 24-hour cycle, while allowing participants to follow their normal routine as far as possible. Ethnic differences in fasting and 24-hour mean concentrations, the rhythmicity and timing of peaks, and nadirs of variables were tested. The relationships between PTH and BTMs were explored.

## Subjects and Methods

### Study design, participants, and sample collection

This study was conducted in three ethnic groups in the environment of their origin and long-standing residency through three research centers: Medical Research Council (MRC)-Human Nutrition Research (HNR), Cambridge, United Kingdom; MRC Keneba, The Gambia; and Shenyang Medical College (SMC), Shenyang, People's Republic of China. Cambridge (latitude 52°N) is an affluent university town with a temperate climate. Keneba (13°N) is a sustenance farming village in central Gambia with a tropical climate with a wet and dry season. Shenyang (42°N) is an industrial city in northern China with a continental climate. The groups have different food patterns, with high calcium intakes from dairy in the United Kingdom, low calcium intakes in The Gambia, and moderate intakes predominantly from green leafy vegetables in China. Levels of physical activity are high and body mass index (BMI) is low in The Gambia compared to the two other groups (7–9, 26). Studies in the United Kingdom and China were performed in winter, when there is no cutaneous vitamin D synthesis, and studies were performed in the dry season in The Gambia, where there is no seasonal variation in vitamin D status. Participants were recruited from the community through advertisement in the United Kingdom and China and by field workers in The Gambia after an initial screen on eligibility through the Kiang West Demographic Surveillance database. The studies were approved by the Cambridge Local Research Ethics Committee, the Gambian Government/MRC Laboratories Joint Ethics Committee, and the Shenyang Medical College Ethics Board. The research was performed in accordance with the Declaration of Helsinki. All potential participants received detailed written and verbal information in their native language. Informed consent was given by all participants.

Participants were 91 healthy British, Mandinka, and Han-Chinese men and women aged 60–75 years (30 or 31 per ethnic group, with an approximate equal split by gender). Individuals were excluded if they were known to suffer from any pathological disorder known to alter calcium or bone metabolism as described before (7). Participants were additionally excluded if they suffered from an irregular sleep pattern and if they had an abnormal hemoglobin (<10g/dL) or plasma creatinine (>115 μmol/L).

The study comprised a 24-hour period during which participants were encouraged to follow their normal routine as far as possible, including meal times and food choices, activities, and wake/sleep times. This was facilitated by community visits in the United Kingdom and The Gambia (at their home, workplace, or location of social commitment). In the United Kingdom, early morning samples and overnight collections took place in the research center. In The Gambia, only early morning collections took place at the research center; the remaining collections took place in the community because the subjects are unaccustomed to an environment with electrical lights and sleeping in separate rooms from their family members. In China, participants stayed in the research center and its vicinity throughout the day because local regulations did not allow for community visits. During stays at the respective research centers, participants were hosted in a “volunteer suite” and had access to recreational facilities.

**Figure 1.**
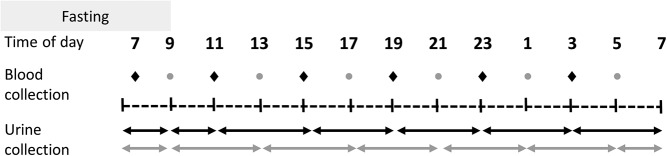
Schematic representation of the study design. Blood and urine samples were collected over a 24-hour period at predetermined time points as indicated by the dots (blood) and arrows (urine). The sample collection schedule was staggered between two subgroups; each participant provided six blood samples to provide 12 collection time points on a group level.

Blood and urine collections took place at 4-hour intervals as indicated in Figure 1, except for the early morning fasting blood and urine sample at the start of the study day. Participants were randomly allocated to one of two groups (A or B), which were staggered by 2 hours, providing 12 time points per 24 hours per group. Blood and urine samples were collected and processed as described before (7). Anthropometry was performed during fasting using standardized techniques (7).

### Biochemical analysis

All assays except PTH (between-assay coefficient of variation, 3.1%) were performed in duplicate. Any samples with a difference in duplicates ≥10% were repeated. All samples from The Gambia and samples for analysis of procollagen type-1 N-propeptide (P1NP), PTH, and 1,25(OH)_2_D from China were transported on dry ice to the MRC-HNR for analyses. The remaining analyses of samples from Chinese participants were performed at SMC using the same methodology and controls as those used at MRC-HNR. Cross-calibrations were conducted for those assays that were performed at different centers by using common controls. The differences between the laboratories were within the supplier's acceptable ranges and those given for the between-assay variation. Therefore, no calibration to a common standard was deemed necessary.

Sample analyses were performed as described before (26). In short, C-terminal telopeptide of type I collagen (CTX) was measured by ELISA (Immunodiagnostics System PLC). Total P1NP and 1,25(OH)_2_D were measured by RIA (IDS Ltd) and UniQ P1NP (Orion Diagnostica Oy). N-mid osteocalcin (OC), bone alkaline phosphatase (BAP), and 25-hydroxyvitamin D [25(OH)D] were measured by chemiluminescence immunoassay (Liaison; DiaSorin). PTH was measured with a chemiluminescence technique (Immulite; Siemens Healthcare Diagnostics). Calcium, phosphate, and albumin were measured by spectrophotometry (Kone 20i; Labsystems), and albumin-adjusted plasma calcium (CaAlb) was calculated as before (26). Between- and within-assay coefficients of variation were as described previously (26). Assay performance was monitored using kit and in-house controls, and performance was within acceptable limits. External quality assessment was obtained through participation in the UKNEQAS Service (www.ukneqas.org.uk) and DEQAS (www.deqas.org) for plasma 1,25(OH)_2_D and 25(OH)D and was traceable to National Institute of Standards and Technology standards for 25(OH)D.

### Data analysis and presentation

A sample size calculation was conducted using cross-sectional data derived from a study with similar populations (7) and was based on a detectable difference between plasma concentration of PTH at the peak and nadir of the 24-hour profile as tested by paired comparison. The mean and SD of the ln-transformed fasting plasma concentration of PTH (nanograms per milliliter) was between 3.5 ± 0.34 (United Kingdom) and 3.8 ± 0.28 (The Gambia). Based on these data, with a significance level of 5% and a power of 80%, an 8% difference would be detectable with a group size of 15 per gender. Therefore, the study included 90 participants (n = 30 per ethnic group).

Non-normally distributed data were transformed to natural logarithms. Data are presented as the fasting mean (SD) or the geometric mean (95% confidence interval [CI]) as based one time point per participant and as the 24-hour rhythm-adjusted mean derived from Fourier regression (geometric mean [95% CI] as derived from six time points per participant). Differences between ethnic groups were tested using one-way ANOVA, with post hoc Scheffé tests.

**Figure 2.**
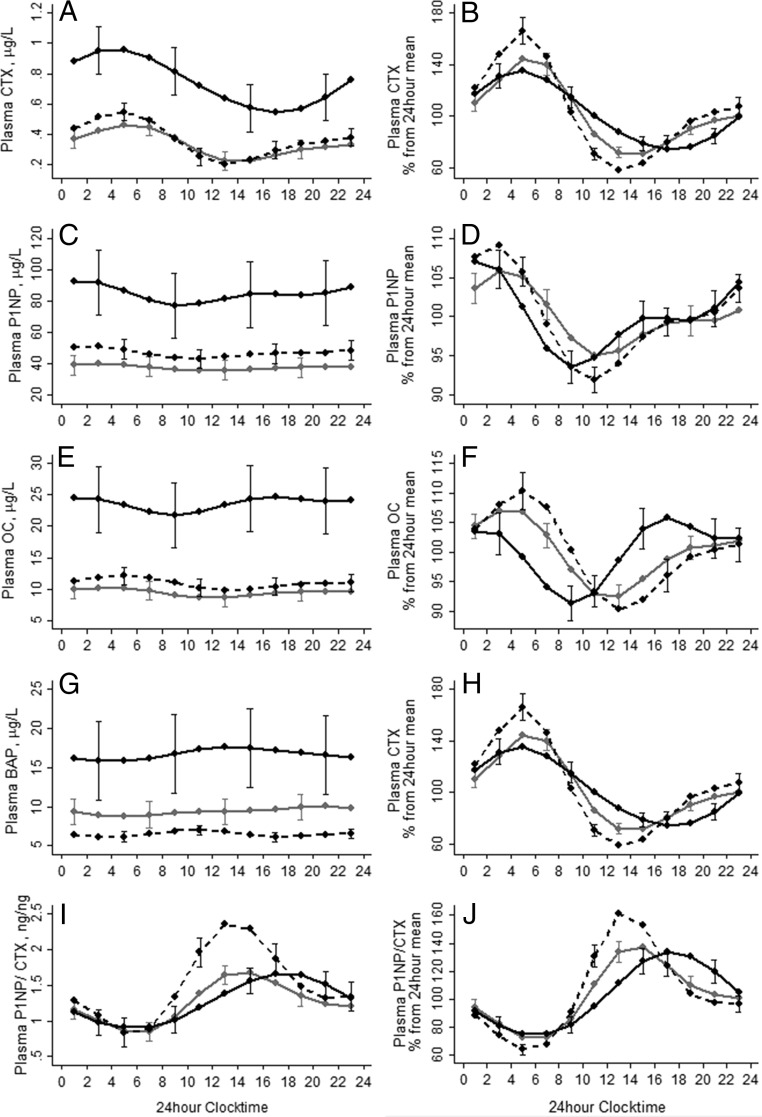
The DR of plasma CTX (A and B), P1NP (C and D), OC (E and F), BAP (G and H), and P1NP/CTX ratio (I and J) in British (gray lines), Gambian (solid black lines), and Chinese (dashed black lines) older adults. The graphs on the left represent data in their units of measurements, and the graphs on the right represent data expressed as a percentage from the 24-hour mean. The lines represent the fitted mean values following Fourier regression, the bars represent 2 × the upper and lower standard error. For ease of reading, standard errors are given for one of three of time points in alternating order for each group.

Fourier (trigonometric) regression was used for the analysis of rhythmicity of data as previously described (28). A detailed description is given in the Supplemental Data. Graphs show the fitted absolute inverse logged mean values (± 2 SD) derived from Fourier regression (Figure 2, three left panels; Supplemental Figure 1) and expressed as a percentage from the 24-hour mean. The latter was calculated to facilitate comparisons of DRs of variables whose absolute mean concentrations differed substantially between groups. The significance of the rhythm was determined by testing the null hypothesis that all Fourier coefficients were zero. The effect size of the rhythm was expressed as the mean (95% CI) coefficient of cyclic variation (CCV). This was calculated as the square root of half the sum of the squared coefficients of the Fourier terms (29). We identified the peaks and nadirs of each fitted diurnal pattern by finding the points at which derivative (with respect to time) of the fitted curve equaled zero using the Newton-Ralphson method. A Monte Carlo sampling method was used to calculate the standard error for the estimates of the timing of the peak or nadir (further details are provided in the Supplemental Data). Differences in the timing of the peaks between groups were tested with the z-test.

Cross-correlational analysis was performed to determine the relationship between the DRs of two variables within each ethnic group (19). Because cross-correlation analysis is likely to show a correlation between any two variables that exhibit rhythmicity, analysis was only conducted with variables between which a relationship is biologically plausible and meaningful (eg, between plasma PTH and CTX, but not calcium, because the feedback loop between these variables is seconds rather than hours). *P* values are given for the lag yielding the strongest cross-correlation. A significant *P* value indicates similarities in the Fourier predicted patterns. Linear regression was used to assess the relationship between plasma concentrations of PTH and BTMs and between CTX and P1NP. Statistical analyses were performed using both early morning fasting data and the 24-hour mean. An interaction term between ethnicity and the dependent variable was used to investigate group differences in the slope and intercept of relationships.

There were significant differences between men and women in 24-hour mean concentrations of the BTMs similar to reports elsewhere (7). However, the CCVs and the timing of the peaks and nadirs of the rhythms were not different for most markers between male and female participants within each ethnic group, and therefore the data for each group are presented with genders combined.

## Results

Groups were well balanced with respect to age and gender frequency (Table 1). British participants were significantly heavier and taller and had a higher BMI and calcium and phosphate intake compared to Gambian and Chinese participants. Gambian participants had higher fasting concentrations of plasma CTX, P1NP, OC, BAP, PTH, 25(OH)D, and 1,25(OH)_2_D than British and Chinese participants and a lower P1NP/CTX ratio compared to Chinese. There were no differences in fasting markers between British and Chinese subjects other than CaAlb and albumin. Group differences in CaAlb were related to their albumin concentrations and were not reflected in ionized calcium (group difference *P* > .05; data not shown).

**Table 1.** Characteristics of Participants: Age, Weight, Daily Dietary Intakes of Calcium and Phosphate, and Plasma BTMs and Regulatory Factors in Early Morning Fasted Samples

	British	Gambian	Chinese
Participant characteristics			
Age, y	65.5 (3.9)	66.1 (4.3)	64.4 (3.5)
Gender, female/male	14/16	17/14	14/16
Weight, kg	79.1 (73.3, 85.4)^G,a C,a^	56.1 (53.1, 59.2)^C,b^	62.9 (60.1, 65.9)
Height, m	1.72 (0.11)^G,a C,b^	1.62 (0.10)	1.64 (0.08)
BMI, kg/m^2^	26.9 (25.0, 28.9)^G,a C,b^	21.4 (10.5, 22.4)	23.0 (22.1, 24.0)
Calcium, mg/d	1076 (961, 1204)^G,a C,a^	282 (240, 333)^C,a^	546 (467, 638)
Phosphate, mg/d	1432 (1312, 1568)^G,a C,a^	705 (638, 778)^C,a^	1158 (1084, 1237)
Fasted morning plasma samples			
CTX, μg/Lψ	0.38 (0.32, 0.46)^G,a^	0.80 (0.69, 0.94)^C,a^	0.42 (0.36, 0.47)
P1NP, μg/Lψ	34.6 (29.7, 40.4)^G,a^	69.0 (59.1, 80.5)^C,a^	42.8 (37.8, 48.5)
OC, μg/Lψ	8.8 (7.6, 10.2)^G,a^	19.3 (16.3, 23.0)^C,a^	10.8 (9.5, 12.3)
BAP, μg/Lψ	8.4 (7.2, 9.7)^G,a^	14.2 (11.9, 16.9)^C,a^	6.6 (6.0, 7.3)
P1NP/CTX, ng/ngψ	0.90 (0.80, 1.00)	0.85 (0.78, 0.92)^C,b^	1.01 (0.92, 1.11)
PTH, ng/Lψ	47.5 (40.9, 55.2)^G,a^	72.0 (62.8, 82.6)^C,b^	55.6 (48.7, 63.6)
1,25(OH)_2_D, pmol/Lψ	124 (110, 140)^G,a^	185 (172, 201)^C,a^	122 (109, 137)
25(OH)D, nmol/Lψ	36.6 (30.3, 44.1)^G,a^	58.6 (52.4, 65.4)^C,a^	31.9 (27.2, 37.5)
CaAlb, mmol/Lψ	2.38 (2.36, 2.40)^G,b C,a^	2.34 (2.30, 2.37)^C,a^	2.14 (2.12, 2.17)
Alb, g/L	37.9 (2.4)^G,a C,a^	32.9 (2.5)^C,a^	43.9 (2.4)
P, mmol/L	1.05 (0.12)	1.03 (0.17)	0.99 (0.17)

Abbreviations: Alb, albumin; P, plasma phosphate. G and C indicate differences compared to British for Gambian (G) or Chinese (C) participants. For normally distributed data, results are expressed as mean (SD); for skewed data (denoted by ψ), the results are expressed as geometric mean (95% CI). Group differences are tested using a one-way ANOVA with post-hoc Scheffé test, on logged data for skewed variables: ^a^, *P* < .01; ^b^, *P* < .05.

**Figure 3.**
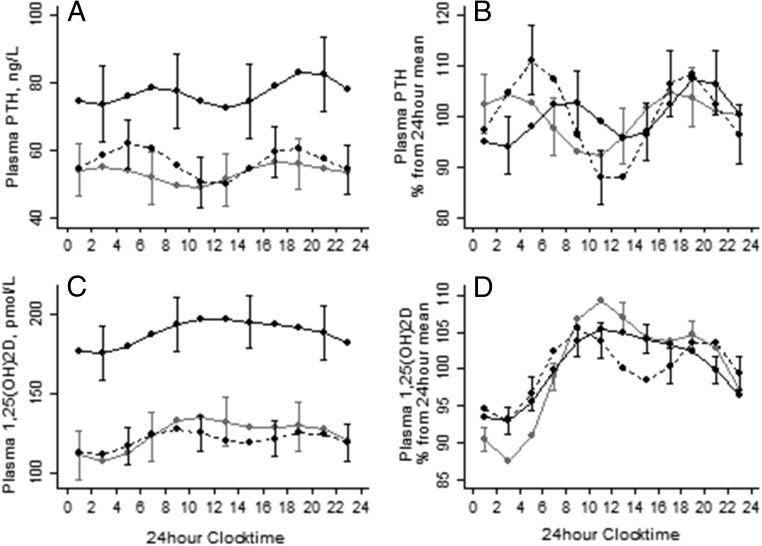
The DR of plasma PTH (A and B) and 1,25(OH)_2_D (C and D) in British (gray lines), Gambian (solid black lines), and Chinese (dashed black lines) older adults. For further details see Figure 2 and Table 1.

Results of the Fourier regression are shown in Table 2, Figures 2 and 3, and Supplemental Figure 1. Similar to fasting values, the 24-hour mean concentrations of plasma CTX, P1NP, OC, BAP, PTH, and 1,25(OH)_2_D were higher in Gambian participants than in British and Chinese participants (Figures 2 and 3, left panels). The 24-hour mean P1NP/CTX ratio in both British and Gambian participants was lower compared to Chinese. Both CaAlb and phosphate were higher in British and Gambian compared to Chinese participants.

**Table 2.** Parameters of the DR of Plasma CTX, P1NP, OC, BAP, P1NP/CTX, PTH, 1,25(OH)_2_D, Calcium, and Phosphate

	British	Gambian	Chinese
CTX			
24-h mean, μg/L	0.33 (0.28, 0.38)^G,a^	0.74 (0.60, 0.88)^C,a^	0.36 (0.31, 0.41)
Significance of rhythm, *P*	.001	.001	.001
CCV, %	23 (20, 26)^C,a^	21 (17,24)^C,a^	32 (29,35)
Peak, 24-h clocktime	5.7 (5.2, 6.3)	4.7 (3.3, 6.1)	5.1 (4.7, 5.5)
Nadir, 24-h clocktime	14.1 (13.5, 14.6)^G,a C,a^	17.4 (16.0, 18.8)^C,a^	13.3 (12.9, 13.7)
P1NP			
24-h mean, μg/L	37.6 (31.5, 43.7)^G,a^	84.5 (64.3, 104.6)^C,a^	46.8 (40.7, 52.8)
Significance of rhythm, *P*	.001	.001	.001
CCV, %	3.3 (2.4, 4.2)^C,a^	4.0 (2.9, 5.1)	5.2 (4.4, 6.1)
Peak, 24-h clocktime	3.7 (2.8, 4.6)^G,a^	1.6 (0.8, 2.4)	2.6 (1.8, 3.5)
Nadir, 24-h clocktime	11.8 (10.5, 13.0)^G,a^	9.3 (8.4, 10.2)	10.6 (9.6, 11.6)
OC			
24-h mean, μg/L	9.4 (8.0, 11.0)^G,b^	23.6 (18.5, 28.7)^C,b^	10.9 (9.0, 12.3)
Significance of rhythm, *P*	.001	.001	.001
CCV, %	4.7 (3.7, 5.6)	4.7 (3.1, 6.3)	6.2 (4.8, 7.6)
Peak, 24-h clocktime	3.7 (2.7, 4.7)^G,b C,b^	1.3 (20.7, 5.7)^C,b^	5.0 (3.9, 6.1)
Nadir, 24-h clocktime	12.1 (11.2, 13.1)^G,b C,b^	9.2 (8.1, 10.3)^C,b^	13.3 (12.5, 14.1)
BAP			
24-h mean, μg/L	9.4 (7.8, 10.9)^G,a^	16.6 (11.7, 21.5)^C,a^	6.5 (5.9, 7.0)
Significance of rhythm, *P*	.001	.001	.003
CCV, %	4.5 (2.8, 6.2)	4.3 (3.1, 5.6)	4.8 (2.7, 6.9)
Peak, 24-h clocktime	20.4 (19.2, 21.6)^G,a C,a^	13.1 (9.7, 16.6)^C,a^	10.6 (9.6, 11.6)
Nadir, 24-h clocktime	4.3 (1.8, 6.8)	3.9 (2.9, 4.9)	4.0 (2.8, 5.1)
P1NP/CTX ratio			
24-h mean	1.24 (1.14, 1.34)^C,a^	1.26 (1.13, 1.40)^C,a^	1.50 (1.35, 1.65)
Significance of rhythm, *P*	.001	.001	.001
CCV, %	20 (18, 23)^C,a^	20 (17, 24)	28 (25, 31)
Peak, 24-h clocktime	14.4 (13.9, 14.8)^G,a^	17.4 (16.1, 18.9)^C,a^	13.6 (13.2, 14.0)
Nadir, 24-h clocktime	6.1 (5.5, 6.6)	5.9 (4.5, 7.2)	5.5 (5.1, 6.0)
PTH			
24-h mean, ng/L	53.4 (46.4, 60.4)^G,a^	77.1 (67.3, 86.9)^C,a^	56.6 (50.1, 63.1)
Significance of rhythm, *P*	.013	.024	.001
CCV, %	4.1 (1.3, 6.9)	4.1 (1.2, 7.0)	7.4 (4.4, 10.4)
Peak 1, 24-h clocktime	17.5 (15.4, 19.6)^G,a^	19.9 (18.1, 21.6)	18.4 (17.3, 19.6)
Peak 2, 24-h clocktime	3.3 (1.0, 5.6)^G,b^	8.0 (5.8, 10.2)	5.4 (4.4, 6.4)
Nadir 1, 24-h clocktime	22.4 (20.0, 0.7)	2.3 (0.7, 3.8)	23.7 (22.5, 00.9)
Nadir 2, 24-h clocktime	10.3 (8.8, 11.9)	13.6 (12.0, 15.3)	11.9 (11.0, 12.8)
1,25(OH)_2_D			
24-h mean, pmol/L	124 (109, 139)^G,a^	188 (172, 204)^C,a^	120 (109, 131)
Significance of rhythm, *P*	.001	.001	.001
CCV, %	7.1 (6.2, 8.0)^G,a C,a^	4.4 (3.4, 5.3)	3.7 (2.6, 4.8)
Peak, 24-h clocktime	10.9 (10.4, 11.4)	12.1 (7.9, 16.2)	9.3 (8.5, 10.0)
Nadir, 24-h clocktime	3.0 (2.6, 3.4)	2.3 (1.4, 3.0)	2.5 (1.8, 3.2)
CaAlb			
24-h mean, mmol/L	2.40 (2.38, 2.42)^G,b C,a^	2.36 (2.34, 2.39)^C,a^	2.12 (2.11, 2.14)
Significance of rhythm, *P*	.003	.02	>.05
CCV, %	0.6 (0.1, 0.11)	0.7 (0.2, 0.12)	Undetectable
Peak, 24-h clocktime	12.9 (12.1, 13.7)	15.4 (12.2, 18.7)	Undetectable
Nadir, 24-h clocktime	5.7 (4.9, 6.6)	8.8 (7.9, 9.8)	Undetectable
Phosphate			
24-h mean, mmol/L	1.12 (1.09, 1.16)^C,a^	1.10 (1.05, 1.15)^C,a^	0.98 (0.93, 1.03)
Significance of rhythm, *P*	<.001	<.001	.003
CCV, %	5.2 (4.1, 6.2)	6.0 (4.7, 7.3)	6.0 (4.0, 7.9)
Peak, 24-h clocktime	2.48 (1.92, 3.03)^C,b^	1.67 (22.8, 2.5)	3.45 (2.7, 4.2)
Nadir, 24-h clocktime	9.89 (9.37, 10.4)	11.0 (9.97, 12.03)	10.5 (9.65, 11.31)

Data are expressed as mean (95% CI). Ethnic differences in the 24-hour mean are tested using ethnicity as an independent categorical variable in regression. Ethnic differences in the CCV% and the timing of the peak(s) and nadir(s) are tested using the z-test. ^a^ *P* < .01; ^b^ *P* < .05. G and C indicate differences for the Gambian (G) and Chinese (C) participants compared to British participants.

In all groups, the DR was significant for all measured plasma markers, except for CaAlb in the Chinese group. Despite the pronounced differences in absolute BTM and PTH concentrations between the British and Gambian groups, the %CCVs of these markers were similar (Table 2); this was also reflected when data were expressed as the percentage of the 24-hour mean (Figures 2 and 3, right panels). In contrast, compared to the British and Gambian groups, %CCVs of CTX, P1NP, and P1NP/CTX were higher in the Chinese group. The British group had a higher %CCV for 1,25(OH)_2_D. The %CCV for CaAlb was very low in all groups

There were group differences in the timing of the peaks of most BTMs and PTH (Table 2 and Figures 2 and 3). However, all groups exhibited a unimodal pattern for all BTMs and phosphate with an early morning peak and lower concentrations during daytime, with the exception of BAP. PTH exhibited a bimodal pattern in all groups.

**Figure 4.**
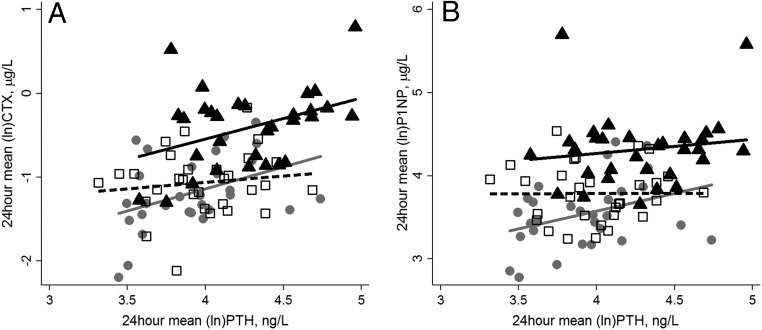
The relationship between the 24-hour mean concentration of PTH and CTX (A) or PTH and P1NP (B) in British (B, gray solid line and circles), Gambian (G, black solid line and triangles), and Chinese (C, black dashed line and open squares) older adults. The regression equations for the different groups were: 24-hour mean (ln) CTX—for B, −3.23 + 0.53 * (ln)PTH; R^2^ = 0.16, *P* = .03; for G, −2.51^B^ + 0.49 * (ln)PTH; R^2^ = 0.15, *P* = .03; for C, −1.44^B^ + 0.08 * (ln)PTH; R^2^ = 0.007; *P* = .6; 24-hour mean (ln) P1NP—for B, 1.87 + 0.42 * (ln)PTH; R^2^ = 0.12, *P* = .6; for G, 3.57^B^ + 0.17 * (ln)PTH; R^2^ = 0.02, *P* = .4; for C, 3.77 + 0.002 * (ln)PTH; R^2^ = 0.0001, *P* = .9. ^B^ Indicates significant difference from British participants in the intercept.

In all groups, there was a significant cross-correlation between the DRs of PTH and both CTX (all *P* < .05) and P1NP (all *P* < .05) and of PTH with 1,25(OH)_2_D and phosphate (all *P* < .05). Examination of the relationships between fasting (data not shown) and the 24-hour mean plasma concentrations of PTH and CTX showed a similar positive relationship for older British and Gambian adults; this was significant when the 24-hour mean values were used. This relationship was not found in Chinese participants. There was no significant relationship between PTH and P1NP in either fasting (data not shown) or 24-hour mean values in any of the three groups, although the relationship was approaching significance in British participants (Figure 4). There was a highly significant correlation between CTX and P1NP for all groups (all *P* < .001) for both fasting concentrations (R^2^ = 0.64, 0.73, and 0.53 for the UK, The Gambia, and China, respectively) and the 24-hour mean (R^2^ = 0.71, 0.75, and 0.58 for the UK, The Gambia, and China, respectively). There was no significant difference in either the intercept or the slope in the CTX and P1NP relationship between groups.

## Discussion

In this three-country study in older people, bone turnover, PTH, and 1,25(OH)_2_D exhibited a significant DR, irrespective of ethnicity and the associated differences in absolute plasma concentration of BTMs, PTH, and 1,25(OH)_2_D. The unimodal pattern of the DR of CTX, P1NP, 1,25(OH)_2_D, and phosphate and the bimodal pattern of PTH were similar between groups and to those described in other populations that were predominantly white (16, 30, 31). The DRs of CaAlb were consistently low. In addition, despite pronounced differences in lifestyle (eg, meal times and sleep/wake patterns), the timing and CCV% of the BTMs were remarkably similar between groups, in particular the British and Gambian groups. There were relatively strong cross-correlations between the DRs of PTH and the BTMs and with 1,25(OH)_2_D in all groups, indicating that the rhythmicity of BTMs shares a degree of similarity with those of the regulatory hormones, suggesting that these hormones at least in part drive the process of bone remodeling. Jointly, these data suggest that environmental factors do not strongly influence the DRs and that they have a truly endogenous circadian component that is conserved independently of external stimuli. Also, the absolute plasma concentrations of PTH and 1,25(OH)_2_D, which are likely influenced by population differences in calcium intakes, did not appear to influence the degree of diurnal rhythmicity of bone metabolism as reported in other studies (21, 32, 33). This might indicate that alterations of the rhythmicity of PTH, bone resorption and formation, and the subsequent loss of coupling of bone resorption and formation, which may be associated with a continuously elevated PTH, may occur at ranges of PTH that differ between populations.

In all groups, there was a very strong positive association between the markers of bone formation and resorption, albeit at different concentration ranges, indicating a high degree of coupling. In the British and Gambian groups, there was also a significant positive association between PTH and bone resorption as estimated by plasma CTX. This was not observed in the Chinese group, similar to the absence of a relationship between PTH and BMD in this group (11). These findings suggest that there may be population differences in the PTH-dependent and -independent components of the regulation of bone resorption (22).

The etiology of the DR of bone resorption is not fully understood. It has been shown to be independent of gender, age, and menopausal status in healthy adults (16) and to persist after 5 days of bed rest (16). The DR of plasma CTX was reported to be altered by fasting or feeding (34) and was thought to explain the nocturnal peak in bone resorption (18). Despite differences in the composition of the habitual diets, meal times, and other factors between older British, Gambian, and Chinese adults (7) influencing the fasting and overall 24-hour mean concentrations of BTMs, this did not appear to influence the timing of the nocturnal peaks of CTX or P1NP, which were similar between groups and compared to several previous reports in men and pre- and postmenopausal Western women (14, 16, 35). The independence of the diurnal and circadian rhythm of BTMs of environmental factors was also reported in in vitro and experimental animal models (24).

The diurnal amplitude of bone resorption marker CTX was greater than that of markers of bone formation in all three ethnic groups, which is consistent with previous reports in Western populations (14, 17, 36). This may relate to differences in the metabolism or half-lives of the markers, CTX having a relatively short half-life of approximately 1 hour (37), whereas P1NP and BAP have a plasma half-life of approximately 10 hours and 1.7 days, respectively (38). In addition, differences in the physiological function of these markers (which are still largely unknown) may explain differences in the peak time observed.

The P1NP/CTX ratio may be used as an indicator of the balance of bone formation to bone resorption. A low P1NP/CTX ratio is thought to correlate with net bone loss, but this still needs to be validated by histomorphometry (39). The data suggest that the P1NP/CTX ratio is lower at night in all three ethnic groups, which is likely explained by the larger nocturnal increase in CTX compared to P1NP. This supports the suggestion that there is net bone resorption at night and net bone formation during the day (17), which has potential clinical implications. Eastell et al (23) found that the nocturnal increase in urinary deoxypyridinoline/urinary creatinine excretion was greater in osteoporotic women (+62%) compared to normal women (+48%). Our data showed, however, that these markers may have their peaks and nadirs occurring at different times. Therefore, the evaluation of the ratio at any one time point may give a false representation of their daily proportions. It is likely that the ratio of the 24-hour mean of P1NP and CTX is a more accurate measure of the coupling of bone formation and bone resorption.

Limitations of this study are the small sample size and the limited number of time points of blood collection, limiting the detailed investigation of rhythmicity of variables. We only performed studies during one season; differences between groups may depend on factors influenced by season, such as vitamin D status, availability of foods, and length of day. In addition, in the absence of normative data on normal and osteoporotic ranges of BMD for all three ethnic groups, we did not collect data on bone density or bone loss in the years preceding this study. We can therefore not exclude that this study included participants with subclinical or undiagnosed abnormalities in bone metabolism.

In conclusion, this study demonstrated a significant DR for markers of bone formation and bone resorption in older adults from the United Kingdom, The Gambia, and China. Despite differences in 24-hour mean plasma concentrations and known lifestyle and environmental differences between these groups, diurnal patterns of BTMs were remarkably similar between ethnic groups and to those described in white populations. This may indicate an innate circadian component that underlies these DRs, which is conserved independent of external stimuli. Group differences in overall 24-hour mean concentrations may reflect lifestyle differences, such as the low dietary calcium intake in older Gambian adults, which acts to stimulate PTH secretion, leading to an increase in both bone formation and resorption. This indicates that alteration of rhythmicity associated with an elevated PTH and the associated loss of coupling of bone resorption and formation reported in other studies do not uniformly occur in different populations. In addition, the association between PTH and markers of bone turnover differed by ethnic group. This needs to be considered in the interpretation of PTH as a risk factor of increased bone turnover and loss across populations.
